# Consequences of COVID-19 Outbreak in Italy: Medical Responsibilities and Governmental Measures

**DOI:** 10.3389/fpubh.2020.588852

**Published:** 2020-12-08

**Authors:** Giovanna Ricci, Graziano Pallotta, Ascanio Sirignano, Francesco Amenta, Giulio Nittari

**Affiliations:** ^1^Scuola di Giurisprudenza, Università di Camerino, Camerino, Italy; ^2^Scuola di Scienze del Farmaco e dei Prodotti della Salute, Università di Camerino, Camerino, Italy

**Keywords:** health emergency, Italy, government measures, bonus, medical responsibility, COVID-19

## Abstract

The COVID-19 pandemic has shocked the world causing more victims than the latest global epidemics such as Severe Acute Respiratory Syndrome Coronavirus (SARS-CoV) in 2003, and the Middle East Respiratory Syndrome Coronavirus (MERS-CoV) in 2012. Italy has been one of the most affected countries, and it had to deal with an already weak economic condition and cuts to public health services due to budgetary requirements from the last decade—something that made the situation even more dramatic. Deaths have exceeded 600.000 worldwide. During the emergency, regulatory measures were taken to counter the situation. This study highlights the main anti-COVID-19 government measures to support doctors and healthcare professionals, and it analyzes how to respond to the many requests complaining about neglectful healthcare professionals during the spread of the infection. For all those healthcare workers who died on duty, a compensation plan is assumed through a solidarity fund. The same solution cannot be granted to all patients, given the difficulty in assessing the responsibility of the doctor not only during an emergency but with insufficient instruments to cope with it as well.

## Introduction

The SARS-CoV-2 outbreak, which has been declared a pandemic by the World Health Organization (WHO) on March 11, 2020, has shocked the world (1). The medical class has been immediately the frontline, called to face a great emergency, worsened by the sudden spread of the infection (2).

In Italy, due to the many cuts to public health services of the past few decades, the emergency has been experienced by healthcare professionals as a sacrifice (3). The public health sector was completely unprepared for such a serious event. As a result, the Italian Government has implemented exceptional urgent regulatory measures to support the population that was quickly sinking into an economic crisis, the most serious one since the Second World War.

The health situation before the pandemic saw the protection of elderly people as a milestone since the aging process of the Italian population was strong and with a low fertility rate (1.29 children per woman) (4).

The pandemic exposed the weaknesses of the Italian health system, probably due to the lack of foresight in the political and socio-health fields. In recent years, action has been taken aiming at economic efficiency and savings, though neglecting the negative effects due to poor investment in health. Investments that could have indirectly generated social and economic benefits, with an increase in people's quality of life (4).

The emergency required the reorganization and increase of resources, which proved to be insufficient. In the initial phases of the pandemic access to treatment could have been diluted, to cope with the very high number of hospitalizations of COVID-19 patients that occurred during the lockdown (4).

This situation could be linked to the decentralized organization of public health in Italy, where the regions can act and face the pandemic with a certain freedom, not always in harmony with each other and with the central government (4).

From 2001 to 2019, the state health-requirement in absolute terms has increased, passing from 71.3 billion in 2001 to 114.5 in 2019. If 10 years ago the 105.6 billion euros were 7% of national wealth, in 2019 114.5 billion was 6.6%: a 0.4% cut in GDP in 10 years, which bears the signature of the governments Berlusconi IV, Monti, Letta, Renzi, Gentiloni, Conte (4).

From the GIMBE Evidence for Health foundation report, in the decade 2010–2019, the public financing of the National Health System increased by 8.8 billion euros, growing on average by 0.9% per year, a rate lower than that of inflation annual average growth of 1.07% (5).

So, it grew in absolute terms, but less than inflation.

Besides, another 37 billion euros had been promised for the National Health System, of which 25 billion in 2010–2015 resulting from cuts due to financial measures, and 12 billion in 2015–2019 for public finance needs. Therefore, fewer resources have been allocated to Healthcare than those planned and calculated based on health requirements (5).

The struggle against the virus took place in the hospitals, with fewer and fewer intensive care beds available and the health personnel forced to overwork. Frequently, medical and health personnel have been stuck in hospitals with heavy work shifts due to the lack of other colleagues. Very often doctors and nurses have been infected and deceased (6).

The Italian government has issued a series of decrees to support the population and the medical class, in such a difficult situation (7, 8).

In this work, only the laws enacted as an emergency response are considered.

These measures are aimed at financially facilitating the population and consist of economic bonuses, reduction or suspension of taxes, credit instruments, and guarantees for businesses and support for family life. Banks and insurance companies have also tried to adapt to the situation by offering new products and services.

## Context

This study reviews the major measures issued by the Italian Government to support the medical class during the emergency (9).

The three decrees of the Prime Minister issued in the heart of the COVID-19 emergency were taken into consideration.

The period taken into consideration begins with the WHO pandemic declaration on March 11, 2020, and ends on June 4, 2020, that corresponds to the gradual re-start of activities.

In this context, the need to evaluate the claims for damage coming from wrongdoing that's reaching the judicial offices has been analyzed.

After these challenging months, the first requests for doctors' professional responsibility are emerging, due to the numerous deaths, especially those that occurred in hospitals and protected residences for the elderly.

These requests are based on the alleged inadequacy of facilities concerning anti-contagion precautions for patients and the elderly—despite the health of these patients are more vulnerable.

Authors agree that, in an emergency such as that caused by SARS-CoV-2, the doctor's professional liability law should become milder and adjusted to the pandemic context.

In case the doctor's responsibility is not attributable, the doctor cannot be blamed for civil and criminal liability, especially in an emergency context. In this emergency, with the inability to perform autopsies, it is difficult to state if the death has been caused by COVID-19 or other concomitant causes.

In this context, the measures taken by the Italian government mainly concerned financial and organizational benefits for all workers in the health sector who found themselves unemployed due to the pandemic.

The aim of the “Cura Italia” and “Liquidity” decrees is to strengthen the human and instrumental resources of the national health service in the fight against the pandemic.

## Discussion

### “Cura Italia” and “Liquidità” Decrees

To help the national health system in this emergency, the Italian Government has issued some decrees (10). The most significant one is the decree called “Cura Italia.” The decree intervenes on four main fronts and other sectorial measures:

1. Funding and helping the national health system with more measures, civil protection and other public entities engaged on the emergency front;
2. Support for workers and employers to protect work and income continuity;
3. Credit support for families and micro-, small- and medium-sized companies, through the Central Guarantee Fund (CGF), (Article 49). The CGF authorizes the financing that the company requests from the banking system. It also intervenes in favor of the “CONFIDIS” (Collective Guarantee Consortium of Loans, which carries out guaranteeing activities to facilitate businesses), taking full responsibility in case the financial agreement is not paid off (8).
4. The suspension of tax payments (Article 62) concerns the income of workers who carry out businesses, art or professional activities with revenues of < €2 million, and for all of those in the so-called “Red areas,” whose income tax deadlines for payments were February 21, March 8, and March 31, 2020 (8).
5. Suspension of payment obligations for taxes and contributions as well as other obligations and tax incentives for the sanitation of workplaces and bonuses for employees who remain in service.

The decrees promise concrete aid to doctors during the SARS-CoV-2 emergency (Table 1). One of the first measures allocates funds for 150 million to pay overtime shifts more to doctors and nurses working in hospitals during the coronavirus outbreak. Among the measures for health personnel, there is the chance to keep on duty also the physicians who are about to retire. The master's degree in medicine and surgery immediately becomes qualifying for the practice of the doctor-surgeon profession, exempting the state exam during the outbreak period. An examination that, according to Italian law, must be passed by a graduate in medicine and surgery before he can operate professionally as a doctor. The state income support provides €600,00 to freelancer medics who have seen their turnover decreased by more than 33% starting from March and for no more than 90 days. In addition to that, there is a bonus of €1.000,00 for physicians, paid by ENPAM, the social security institution for this category (8). Article 22 of the decree extends the lay-offs even to employers that employ even just one employer as study staff. In support of families, the “Cura Italia” decree also provides professionals with the possibility of obtaining a €600.00 bonus for the purchase of baby-sitting services, according to the forced closure of schools. The bonus rises to €1,000.00 for employees of the health sector, public and privates belonging to the category of doctors, nurses, and other health professionals. Furthermore, health professionals who hold a first home loan will be able to request the suspension of its payment if they have experienced a decrease in turnover of more than 33% in a quarter, following February 21, 2020, compared to the last quarter of 2019.

**Table 1:** Summary of the main contents of the “Cura Italia” and “Liquidity” decrees promulgated by the Italian government.

**Decree**	**Health sector**	**Labor sector**
“Cura Italia” (7)	▪ funds for 150 million to pay overtime shifts to doctors and nurses ▪ the master's degree in medicine and surgery becomes qualifying for the practice of the medical profession	▪ 600–1000€ bonus possibility to purchase baby-sitting services due to the forced closure of schools ▪ Possibility to obtain bank loans without guarantees ▪ Suspension of payment obligations for taxes and contributions as well as other obligations and tax incentives for the sanitation of workplaces and bonuses for employees who remain in service ▪ Possibility to renegotiate existing loans ▪ Extension of the tax credit for 50% of the costs of working environments sanitization, and also for the purchase of personal protective equipment
“Liquidità” (8)	▪ The state income support provides €600,00 to freelancer medics who have seen their turnover decreased by more than 33% starting from March and for no more than 90 days	▪ Credit support for families and micro, small- and medium-sized companies, through the Central Guarantee Fund (CGF) ▪ The suspension of tax payments (Article 62) concerns the income of workers who carry out businesses, art or professional activities with revenues of < €2 million, and for all of those in the so-called “Red areas”

For doctors who are included in micro, small and medium-sized enterprises, including individuals, damaged by the COVID-19 epidemic, the decree introduces the suspension of mortgages and other installment loans or leasing until September 30, 2020. Another measure for professionals (including doctors) is the possibility of obtaining bank loans without guarantees, thanks to an agreement with the “Cassa Depositi e Prestiti.” The latter is an Italian Financial Institution controlled by the Ministry of Economy and Finance with the mission of promoting the country's growth and managing postal savings. Up to 80% of the requested amount can be obtained by invoking the public guarantee fund for small and medium-sized enterprises and professionals, or up to 90% through certain credit guarantee agencies (CONFIDI). This possibility can also be used to renegotiate existing loans. Also, self-assessment payments that expire in the period between March 8, 2020 and March 31, 2020 are suspended for individuals carrying out business, art, or other professions. For the same subjects, the decree recognizes “a tax credit” up to 50% of the documented costs of environment sanitizing and working tools up to a maximum of €20,000.00.”

Moreover, the decree of April 8, 2020 guarantee an extension of the tax credit for 50% of the costs of working environments sanitization, and also for the purchase of personal protective equipment, such as face masks and safety goggles (7).

### New Measures for Credit and Insurance

The most important medical federations such as Ente Nazionale di Previdenza ed Assistenza dei Medici e degli Odontoiatri (ENPAM), Federazione Nazionale degli Ordini dei Medici Chirurghi e degli Odontoiatri (FNOMCEO), and Federazione Italiana Medici di Medicina Generale (FIMMG) have decided to set up a working group to strengthen the protection for doctors, currently engaged in the containment of the spread of SARS-CoV-2 (11). This working group will evaluate the chance to extend the protection provided by the National Institute for Accidents at Work (INAIL) to freelance and/or affiliated doctors and dentists (who are not currently benefitting from the insurance coverage of INAIL) (12).

There are also contractual increases for family doctors, pediatricians, and outpatient specialists during the COVID-19 outbreak to allow doctors to equip themselves with technological tools for patient telemonitoring, for a total of 400 million euros.

Even for small-professional doctors, there is a 100% guarantee for new financing, with a maximum duration of 6 years without evaluation procedure, for a maximum amount of €25,000.00. Furthermore, tax deadlines have been extended (13).

Insurance companies are also offering optional services and supplementary policies, recognizing indemnities in case of hospitalization, cash bonuses, special medical visits, teleconsultations, etc. Insurance companies have created *ad hoc* products and supplemented health policies with specific services. Free of charge and temporarily, some companies have extended guarantees and services included in health insurance policies to let insured persons affected by the virus get daily indemnities in case of home quarantine, and compensation in case of hospitalization in intensive care (14).

INAIL has included in the category of accidents at work COVID-19 infections for doctors, nurses and other operators of health facilities in general, employees of the national health system and any other public or private structure insured with the Institute, occurred in the workplace or simply due to the working activity (15).

The public or private company has the task of promptly communicating to the competent authorities the health conditions of its employees regarding a possible coronavirus infection.

The initial term of INAIL protection is the date of contagion confirmation through specific tests. Healthcare workers who are quarantined for public health reasons are excluded unless they are positive. In this case, they are protected for the entire quarantine period and any subsequent time due to protracted illness, which determines temporary incapacity to work. Finally, considering that the infection may occur during home-work travel, the hypothesis of an on-going accident is taken into consideration.

### The Professional Responsibility of the Doctor

Although healthcare professionals are fighting the pandemic with tough work shifts and shortages of staff and resources, several claims for professional liability have been received (15). The epidemic weighs heavily on the performance of healthcare professionals and can give rise to civil liability judgments (contractual and extra-contractual) against them, in all cases in which a medical error can be identified to the detriment of a patient suffering from COVID-19.

For this reason, the federations and national councils of health and socio-health professions, and the Ministry of Health have collected their considerations in a series of proposals to amend the law on the liability of professional doctors (16).

The need for such maneuvers derives from the fact that there are currently no clear regulations establishing the procedures for attributing medical responsibility in case of health emergencies. The current legislation is limited to blaming negligent, superficial, and repetitive conduct.

The main proposals consist of a provision that limits the liability of the health care professional during the emergency period to malicious assumptions—but also, an “evident” prediction of gross negligence, with the restriction of liability to “gross” fault cases. These limitations should be implemented both in civil, criminal, and administrative cases. The aim is to avoid a long judicial involvement to the detriment of doctors, but also to protect all those who, out of a pure spirit of generosity, made themselves available in the emergency even without so much experience, physicians called back into service, or with different specializations (17).

The contexts in which the provision was deemed applicable concern necessarily extraordinary and exceptional cases, as “not adequately studied by science or tested by practice.”

The proposed changes also involve the responsibility of public and private hospitals forced to work with limited resources and by adapting ordinary activities into emergency activities.

It should be mentioned that the health policy choices in Italy have severely penalized healthcare in the past few years, reducing funds dramatically (18).

To give an example, Germany spends €3.600,00 in healthcare per year for each German citizen, while Italy spends €1.800,00. Intensive care beds in Italy in 2017 were 8.5 per 100,000 inhabitants, whilst in Germany, these were 34 in the same period (19).

As for compensation for damage from COVID-19, two groups of recipients can be distinguished: (1) health workers killed by the virus for work reasons, and (2) victims of the pandemic among ordinary citizens. Those who contracted the virus by sacrificing their lives serving the country as doctors, nurses, and pharmacists and all collaborators involved in the coronavirus emergency must be compensated (20).

The hypothetical form would be compensated with a solidarity fund for the victims. Some jurists refer to these victims as the so-called “victims of duty” (21).

The situation is more complex for infected and deceased citizens. Even if the cases were restricted to misconduct or gross negligence, a solidarity fund can hardly be conceived as for health workers for several reasons. First of all, the verification of the fault, which is carried out in the aftermath, will depend on the competence of the doctor, on the objective conditions of the patient, and on the context in which he found himself operating. Farther to the fault, the connection must be ascertained. Something to be equally sure about is the correct behavior the doctor should have had to save the patient. All of this is not easy to determine, as nobody knows exactly about the “behavior” of this virus, and it would be difficult to establish in a court “what would have happened if” the patient had been managed in a way or another. Contributing factors such as advanced age, the frequency of associated diseases also play an important role. Last, but not least, not enough swabs or autopsy have been made. Ergo, it will be difficult to tell the difference between “coronavirus-caused” or “with coronavirus” death.

## Limitations

Some limitations apply to this work. The legislative experience described can be used as a starting point for future measures by other nations. However, this work deals with regulations promulgated in Italy, which has legislation and a national health system that may be different from those of other countries.

The judicial system may also differ, with differences in handling malpractice requests due to the fight against COVID-19.

## Conclusions

The SARS-CoV-2 virus has shocked the world causing more victims than recent similar outbreaks.

A treatment protocol, as well as an effective vaccine, are currently under study, but these have not been identified yet (22).

Italy, the third country after China and Spain to be infected as of May 2020, had to deal with an already weakened economic situation and important cuts in healthcare which immediately made the situation very complex (23).

The health professionals worked in an exemplary manner even without suitable or effective protection measures (24, 25).

The need to recover and face the emergency has prompted the Government to promote an emergency decree (26).

There was no shortage of criticism for these measures, from the accusations of unconstitutionality of the lockdown to the long times for the disbursement of the bonuses to the failure to insert some subjects as beneficiaries of the bonuses. It does not seem possible to limit liability to cases of willful misconduct because, even in an emergency, the possibility of errors is increased. In this outbreak context, there are no established guidelines or good practices, and there are no aids to measure diligence and responsibility (17). The evaluation should be limited to cases of gross negligence by the doctor, willful misconduct, or fault for employer liability. In a scoreboard by the Deep Knowledge Group study which analyzes the capacity, scope, diversity, efficiency, and effectiveness of government measures to provide economic support to citizens and businesses, Italy ranks in the tenth place in the world for resilience (27). The renowned Italian ability to bring out the best during emergencies.

## Data Availability Statement

The raw data supporting the conclusions of this article will be made available by the authors, without undue reservation.

## Author Contributions

GR and GN: conceptualization, data curation, formal analysis, investigation, methodology, and writing. GP: supervision, validation, formal analysis, and writing—review and editing. AS and FA: supervision and validation. All authors contributed to the article and approved the submitted version.

## Conflict of Interest

The authors declare that the research was conducted in the absence of any commercial or financial relationships that could be construed as a potential conflict of interest.
